# P-22. Carbapenem-resistant Gram-negative Bacterial Colonization and Infection in Critically Ill Patients in an Intensive Care Unit of a Tertiary Hospital in Bangladesh

**DOI:** 10.1093/ofid/ofaf695.253

**Published:** 2026-01-11

**Authors:** Gazi Md Salahuddin Mamun, Sanzida Khan, Md Shahinur Rahaman, Md Aminul Islam, Shabrina Sharmin, Dilruba Ahmed, Md Ali Amin Nabin, Aninda Rahman, Syeda Mah-E-Muneer, Gemma Parra, Ashley R Styczynski, Fahmida Chowdhury

**Affiliations:** International Centre for Diarrhoeal Disease Research, Bangladesh icddr,b, Dhaka, Dhaka, Bangladesh; icddr,b, Dhaka, Dhaka, Bangladesh; International Centre for Diarrheal Disease Research, Bangladesh, Dhaka, Dhaka, Bangladesh; icddr,b, Dhaka, Dhaka, Bangladesh; International Centre for Diarrheal Disease Research, Bangladesh, Dhaka, Dhaka, Bangladesh; icddr,b, Dhaka, Dhaka, Bangladesh; International Centre for Diarrhoeal Disease Research, Bangladesh icddr,b, Dhaka, Dhaka, Bangladesh; Directorate General of Health Services, Government of Bangladesh., Dhaka, Dhaka, Bangladesh; icddr,b, Dhaka, Dhaka, Bangladesh; Centers for Disease Control and Prevention, Atlanta, GA, United States, Atlanta, Georgia; Centers for Disease Control and Prevention, Atlanta, GA; icddr,b, Dhaka, Dhaka, Bangladesh

## Abstract

**Background:**

Colonization with multidrug-resistant organisms is associated with higher infection rates in high-risk individuals. This study aimed to assess the burden of carbapenem-resistant Gram-negative bacteria (CR-GNB) colonization among patients in an intensive care unit (ICU) and explore associations with clinical outcomes.

**Figure:**
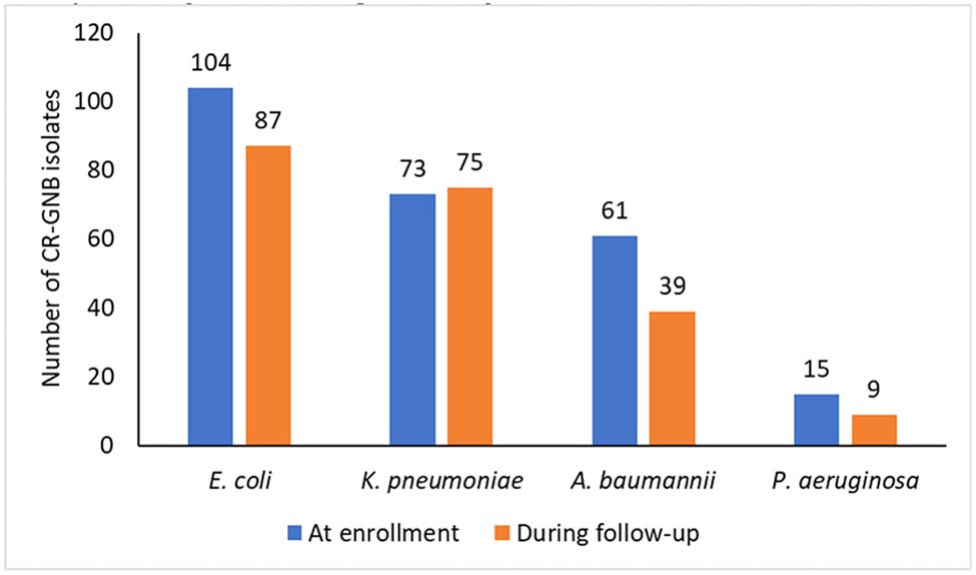
Distribution of colonizing carbapenem-resistant Gram-negative bacterial isolates (CR-GNB) at entry and during ICU stay

**Methods:**

In this prospective cohort study, we enrolled patients within 24 hours of ICU admission at a tertiary hospital in Bangladesh. Patients were assessed for colonization with CR-GNB using rectal swabs collected on enrollment, days 3 and 7, and at weekly intervals until their discharge or death. Bacterial isolates recovered from CHROMagar mSuperCARBA underwent confirmatory identification and susceptibility testing using VITEK-2. Cultures from blood, urine, and tracheal aspirates were performed if infection was suspected. Gram-negative bacteria with resistance to meropenem, imipenem, and/or ertapenem were defined as CR-GNB. Descriptive analysis and log binomial regression were done in Stata v15.

**Results:**

We enrolled 373 patients from July 2023 to January 2024 (median age 40 years; IQR 24-50 years); 63% were male. The median ICU stay was 4 days (IQR: 2-9 days). At enrollment, 135 (36.2%) patients were colonized with at least one CR-GNB; some individuals were colonized with multiple CR-GNB (Figure). Of the remaining 238, 107 (45%) acquired colonization during their ICU stay. Fifty-two (14%) patients had suspected clinical infections, with 29 (56%) having positive blood cultures, including 9 CR-*Acinetobacter baumannii* and 4 CR-*Klebsiella pneumoniae*. Patients colonized with CR-GNB had a higher risk of culture-confirmed infection (RR 1.46, 95% CI 1.26-1.70) and a longer ICU stay (median 6 vs 2 days, p< 0.001).

**Conclusion:**

Colonization with CR-GNB was frequently observed among critically ill patients, many of whom acquired colonization during their hospital stay. Additionally, the significant association between colonization with subsequent infections and increased duration of ICU stay demonstrates the need for enhanced infection prevention and control measures to mitigate nosocomial transmission of CR-GNB and improve patient outcomes.

**Disclosures:**

All Authors: No reported disclosures

